# Development of Chitosan/Bacterial Cellulose Composite Films Containing Nanodiamonds as a Potential Flexible Platform for Wound Dressing

**DOI:** 10.3390/ma8095309

**Published:** 2015-09-18

**Authors:** Fatemeh Ostadhossein, Nafiseh Mahmoudi, Gabriel Morales-Cid, Elnaz Tamjid, Francisco Javier Navas-Martos, Belén Soriano-Cuadrado, José Manuel López Paniza, Abdolreza Simchi

**Affiliations:** 1 Department of Materials Science and Engineering, Sharif University of Technology, PO Box 11155-9161, Tehran, Iran; E-Mails: ostadho2@illinois.edu (F.O.); nafiseh_mahmoudi@mehr.sharif.ir (N.M.); 2 Fundacion Andaltec I+D+i, Poligono industrial Cañada de la Fuente, 23600 Martos, Jaen, Spain; E-Mails: gabriel.morales@andaltec.org (G.M.-C.); navas@andaltec.org (F.J.N.-M.); soriano@andaltec.org (B.S.-C.); jmlopez@andaltec.org (J.M.L.P.); 3 Department of Nanobiotechnology, Faculty of Biological Sciences, Tarbiat Modares University, PO Box 14115-175, Tehran, Iran; E-Mail: tamjid@modares.ac.ir; 4 Institute for Nanoscience and Nanotechnology, Sharif University of Technology, PO Box 11365-9466, Tehran, Iran

**Keywords:** nanocomposite, nanodiamond, chitosan, bacterial cellulose, wound dressing

## Abstract

Chitosan/bacterial cellulose composite films containing diamond nanoparticles (NDs) with potential application as wound dressing are introduced. Microstructural studies show that NDs are uniformly dispersed in the matrix, although slight agglomeration at concentrations above 2 wt % is seen. Fourier transform infrared spectroscopy reveals formation of hydrogen bonds between NDs and the polymer matrix. X-ray diffraction analysis indicates reduced crystallinity of the polymer matrix in the presence of NDs. Approximately 3.5-fold increase in the elastic modulus of the composite film is obtained by the addition of 2 wt % NDs. The results of colorimetric analysis show that the composite films are transparent but turn to gray-like and semitransparent at high ND concentrations. Additionally, a decrease in highest occupied molecular orbital (HOMO) and lowest unoccupied molecular orbital (LUMO) gap is also seen, which results in a red shift and higher absorption intensity towards the visible region. Mitochondrial activity assay using L929 fibroblast cells shows that the nanocomposite films are biocompatible (>90%) after 24 h incubation. Multiple lamellapodia and cell-cell interaction are shown. The results suggest that the developed films can potentially be used as a flexible platform for wound dressing.

## 1. Introduction

Carbon-based nanomaterials, particularly diamond, have recently attracted significant interest due to their promising properties in biotechnology, optics and other materials science fields. Nanodiamonds (NDs) are envisaged as particles that possess exceptional properties including high specific surface area, high chemically inert sp^3^ carbon (diamond) core, surface functionalization capabilities and biocompatibility [1]. A plethora of studies have recently been devoted to the biomedical applications of NDs such as their exploitation in the targeted anti-cancer drug delivery [2], gene delivery [3], antibacterial agents [4], biosensors [5], contrast agents [6], and scaffolds for tissue engineering [7]. Despite the fact that the toxicity of carbon nanomaterials are greatly dependent upon the purity, size, mass and surface functional groups [8], NDs have been shown to be more biocompatible than other carbon nanostructures such as carbon nanotube (CNT) and carbon black [9]. Multifunctional composite materials offer the amenity to achieve the required properties in a single platform [10]. For instance, although ND films prepared by chemical vapor deposition can promote the function of various biological entities and implantable devices [11], their practical applications are limited due to their high rigidity [1]. Therefore, there is an unmet need for the development of composite materials to overcome the mentioned challenge. 

Cellulose is one of the most abundant polysaccharides in nature [12]. While cellulose is mostly derived from plants, a new type of cellulose synthesized by *Acetobacter xylinum* called bacterial cellulose (BC) has been introduced with superior properties such as higher purity [13], surface area [14], crystallinity and moisture retention compared to plant cellulose [15,16]. Biomedical applications of BC have received considerable attention in literature, for example, in wound dressing [17], blood vessels [18], vascular grafts [19] and delivery systems of drug and protein [20,21]. In particular, BC has attracted a host of research interests in skin tissue repair and wound care materials due to its intrinsic nanofibrillated network structure, which closely mimics collagen [15]. Although BC has been shown to be effective as wound dressing, it has no antimicrobial properties by itself to prevent the wound infection [15]. In order to overcome this shortcoming, fabrication of composite blends with other natural biopolymers [17,22] and/or nanoparticles [23,24] have been suggested. Chitosan (CS), the N-deacetylated Chitin derivative, is another natural polysaccharide [25] which has several intrinsic features including antimicrobial activity, biocompatibility, mucoadhesive and hemostatic properties [26,27]. It has been shown that upon degradation, CS is decomposed and releases N-acetyl-β-D-glucosamine leading to fibroblast proliferation and ordered collagen deposition, which ultimately results in faster wound healing process [28].

Therefore, CS/BC composites can potentially be a promising candidate for wound dressing as well as for food packaging. Fernandes *et al.* [29] prepared BC/CS films by solvent casting methods. Their obtained films were highly transparent and flexible with enhanced mechanical properties compared with unmodified CS films. Phisalaphong *et al.* [30] prepared CS/BC blends by adding CS to the culture medium of BC during biosynthesis. They reported improved mechanical properties and water absorption capacity while other features such as water vapor permeation rates, average crystallinity index and anti-microbial ability remained virtually unchanged. Lin *et al.* [31] reported enhanced inhibitory effects of BC/CS films against *Escherichia coli* (*E. coli*) and *Staphylococcus aureus* (*S. aureus*). No adverse effects on *in vitro* cell viability of L929 fibroblast cells were noticed. Animal testing also showed that BC/CS films were more successful at wound closure experiment than BC, commercial Tegaderm hydrocolloid or transparent films. 

Considering the biocompatibility and reinforcing effects of NDs along with the established wound healing properties associated with BC/CS composites, the complementary properties of each component has been utilized to fabricate novel polysaccharide-based composite films containing NDs (up to 4 wt %). The films are transparent and flexible with good biocompatibility to fibroblast cells. The role of NDs is not only to impart mechanical rigidity to the films but also to render potential of controlled drug release (as shown by Lam *et al.* [1]). It is shown that the addition of NDs improves the elastic modulus and thermal stability of the polysaccharide films without hampering *in vitro* cell viability. This research would pave the path for future works to introduce drugs such as anti-cancer chemotherapeutic agents in a flexible and free standing platform capable of promoting wound closure in a short time frame.

## 2. Results and Discussion

### 2.1. Microstructure and Chemical Interactions

**Figure 1:**
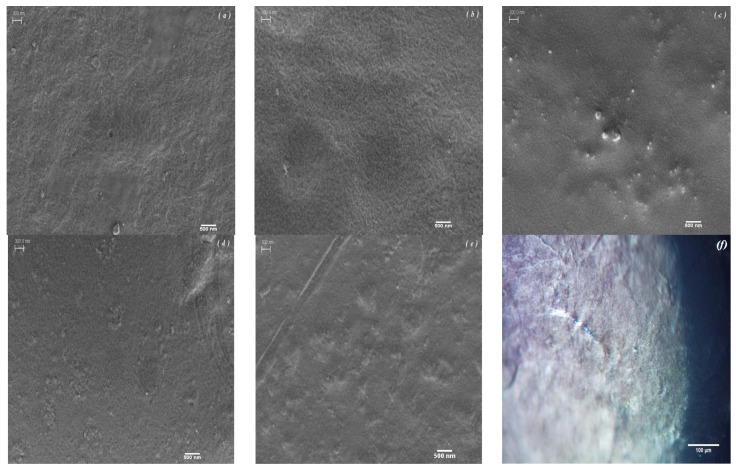
Scanning electron microscopy (SEM) micrographs of the composite films containing (**a**) 0, (**b**) 1, (**c**) 2, (**d**) 3 and (**e**) 4 wt % diamond nanoparticles. The scale bar is 300 nm. (**f**) Transmission optical micrographs of chitosan/bacterial cellulose (CS/BC) film.

Figure 1 shows scanning electron microscopy (SEM) and transmission optical microscopy (TOM) micrographs of the films. According to the published data by the supplier [32], BC possesses a randomly oriented nanofibrillated structure with various pore sizes. This morphology is as a result of glucose polymerization by the bacteria and its secretion to the extracellular matrix, which finally leads to the formation of finely fibrillated web-like structure [33]. Through blending of BC with CS, the structure becomes more densely packed while the fibrillar network of BC is visible (Figure 1a,b). Upon the addition of NDs, no severe agglomeration is seen at low ND loadings (2%), implying the uniform dispersion and wrapping of the NDs particles by the polymer chains. However, at higher concentrations, small clusters are visible that can be due to the high specific surface area of NDs [34]. The transmission optical micrograph (Figure 1f) also reveals that after solvent evaporation, the bacterial cellulose fibrils are actually being embedded in the chitosan films and they preserved their crystalline structure after the fabrication step. This can be inferred from the polarized light birefringence, which is associated with the anisotropic, monoclinic crystal structure of BC [35].

**Figure 2:**
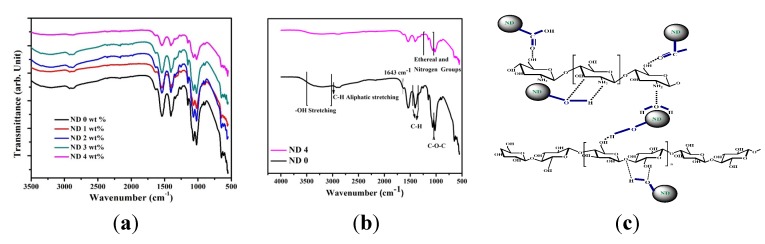
(**a**,**b**) Fourier transform infrared (FTIR) spectra of composite films. (**c**) Schematic illustration of possible interactions between the polymer matrix and functional groups of NDs.

The Fourier transform infrared spectroscopy (FTIR) spectra of CS/BC films containing varying amounts of ND are shown in Figure 2a,b. Due to the similar nature of BC and CS in terms of molecular structure, it is predicable that the two polymers have good miscibility and compatibility [36]. The N–H peaks characteristic of CS molecules overlap the broad absorption shoulder occurring from 3000 to 3500 cm^−1^, which is attributable to –OH stretching vibration [31,35]. The maxima at around 2965 cm^−1^ is assigned to aliphatic C–H stretching vibration [31]. The peak detected at 1643 cm^−1^ comes from the glucose carbonyl of cellulose. The peak at 1610 cm^−1^ is assigned to amide I group in CS. The peaks at 1456 and 1350 cm^−1^ are representative of the symmetric deformation and bending vibration of CH, respectively. The peak at around 1045 cm^−1^ shows the C–O–C stretching vibration. Overall, the results are in good agreement with previous work on CS and BC composite films and firmly verify the presence of many intermolecular hydrogen and ionic bonds as well as a few covalent bonds [30].

The FTIR absorption bands indicate that the incorporation of ND particles in the polymer films does not create or remove new peaks except for some small shifts compared to the CS/BC specimens. Therefore, it is suggestible that no chemical interaction occurs between the polymers and ND functional groups except for the dominant hydrogen bonding [37]. The IR spectra of samples containing ND display broad band at 3410 cm^−1^, which can potentially correspond to N–H stretching [38]. Furthermore, the peak near 1628 cm^−1^ is due to stretching vibration of aromatic sp^2^ carbon bond, which is related to graphite around the ND particles [39]. The band starting from 1000 cm^−1^ with a peak at 1120 cm^−1^ suggests the combination of bands characteristic of nitrogen and the ethereal (≡C–O–C≡) groups. The abundant oxygen containing functional groups on the surface of ND as well as strong van der Waals forces between the high surface area nanoparticles lead to easy agglomeration of ND in the polymer matrix (Figure 1c) [34]. The surface of ND is replete with oxygen containing groups such as hydroxyl and carboxyl, which can interact with the hydroxyl and amine groups of BC and CS. However, the exact ND functional groups are not clearly identifiable although previous attempts have been made to elucidate their nature [40,41]. The potential mode of interaction is illustrated in Figure 2c.

### 2.2. Transparency and Colorimetric Analysis

**Figure 3:**
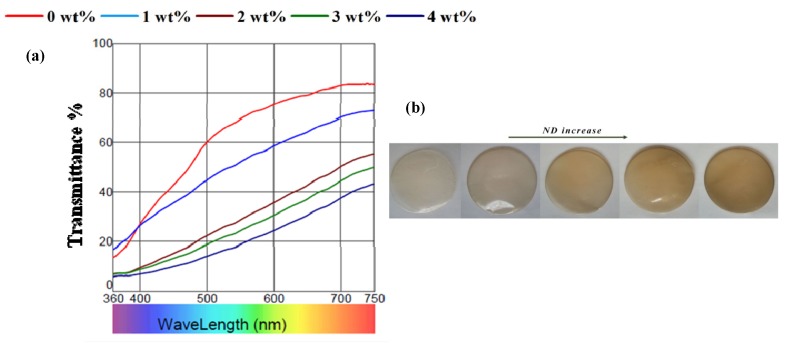
(**a**) Transmission profile of composite films containing different amounts of ND. (**b**) Digital images show the appearance of the composite films.

Figure 3a illustrates the transmission profile in the ultraviolet-visible (UV-Vis) region and digital images of the prepared films, respectively. The results of colorimetric analysis are summarized in Table 1. The CS/BC composite film was considered as transparent (based on L* parameter). With increasing the concentration of NDs, the composite film turn to gray-like and semitransparent, which was indicated by lower whiteness (Lower L*) value, higher redness (higher a*) value and consequently higher total color difference value (∆*E*) of the films. This trend is also evident from the spectra and is due to n → π* transition of the C=O bond. In addition, the BC/CS/ND films have an abundance of highly conjugated aromatic structures from NDs which contribute to π → π* transition. Therefore, there is a decrease in highest occupied molecular orbital (HOMO) and lowest unoccupied molecular orbital (LUMO) energy gap resulting in a red shift and higher absorption intensity towards the visible region [42]. Nevertheless, the colorimetric studies indicated that the homogenous dispersion of ND in the polymer matrix at relatively low concentrations did not impair the transparency. Since light scattering is inversely proportional to the particle size, the nanocomposites remain almost transparent [43].

**Table 1:** Surface color parameters of the examined films. L*, a* and b* correspond to lightness, red/green, and yellow/blue, respectively. In addition, C*, *h*° and ∆*E* represent chroma, hue and color difference with respect to reference, respectively. ND: diamond nanoparticles.

ND%	Thickness (μm)	L*	a*	b*	C*	*h*°	∆*E*
0	3 ± 26	86.00	–0.22	24.04	24.04	90.52	23.22
1	4 ± 28	77.20	3.00	18.45	18.70	80.78	24.21
2	8 ± 30	60.77	6.39	24.4	25.22	75.31	41.33
3	5 ± 23	56.76	6.86	22.56	23.58	73.04	43.96
4	9 ± 27	51.01	7.69	21.82	23.14	70.60	48.92

### 2.3. Thermal Analysis and X-ray Diffraction

**Figure 4:**
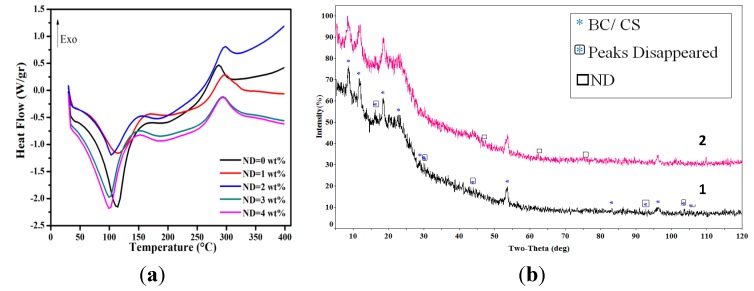
(**a**) Differential scanning calorimetric traces of CS/BC composite films containing different amounts of NDs. (**b**) X-ray diffraction (XRD) pattern of (1) CS/BC and (2) CS/BC/ND (4wt%). Note the disappearance of the green circles and the appearance of purple squares after ND addition.

The differential scanning calorimetric traces of the films are shown in Figure 4a. Three major thermal events can be distinguished, as summarized in Table 2. The first endothermic peak centering on ~100 °C is ascribed to the volatilization of water. The second thermal event, namely the change of slope near the endothermic peak, is a result of local relaxation of the backbone chain of CS [44]. The thermal properties of the films are not changed up to 300 °C; hence, the films are stable enough to be in contact with body or during steam sterilization. The degradation temperature (*T*_d_) of pristine CS and BC is around 250 °C and 320 °C, respectively. The DSC trace for the CS/BC film seems to be a combination of the thermal behavior of BC and CS since the trace follows the “rule of mixtures” (not shown here). The degradation temperature of BC and CS has already been reported to happen at around 300 °C; however, values as low as 270 °C have also been shown based on the different modification of CS [29]. Our results are in concordance with the literature; yet, large batch-to-batch variability is an inevitable factor, which makes the comparison difficult.

**Table 2:** Thermal properties of the examined films.

ND (wt %)	*T*_vap_ (°C)	Enthalpy (J·g^−1^)	*T* (°C)	*T*_d_ (°C)	Enthalpy (J·g^−1^)
0	112.15 ± 0.42	–334.57 ± 1.20	201.72 ±0 .50	286.85 ± 0.76	29.88 ± 4.12
1	114.39 ± 0.25	–213.57 ± 3.02	205.64 ± 0.36	296.34 ± 0.43	100.17 ± 6.71
2	102.86 ± 0.31	–128.48 ± 5.11	191.86 ± 0.87	296.87 ± 0.61	29.98 ± 4.56
3	99.55 ± 0.12	–296.82 ± 2.14	192.87 ± 0.45	292.92± 0 .84	74.44 ± 2.69
4	106.15 ± 0.15	–210.27 ± 1.15	203.10 ± 0.76	288.29 ± 0.26	40.44 ± 3.12

The results also indicate a slight change in the thermal behavior of the CS/BC composite when NDs are added and there is an initial increase (up to 10 °C for ND 2 wt %) in *T*_d_ followed by a decrease (Table 2); yet the overall degradation temperature for the nanocomposite is higher than that of the unmodified films. The decrease in the *T*_d_ value at higher ND contents can be associated with the large surface area to volume ratio of NDs. As this ratio increases, more free volumes would be created in the polymer matrix so as to provide more free space for large polymer chain movements [34]. However, the trend is erratic and cannot be all-inclusive. The reason might be best justified considering that the very same large surface area to volume ratio leads to the self-aggregation of particles, which is not an easily controlled process. Therefore, an increased degradation temperature is expected for the composite films with uniform distribution of NDs, as shown by Morimune *et al.* [39] for polyvinyl alcohol films containing 5 wt % NDs.

Figure 4b illustrates X-ray diffraction (XRD) patterns of the CS/BC films in the absence and in the presence of 4 wt % ND. Diffraction peaks at 16.2° and 22.8° correspond to the (110) and (200) planes of BC, respectively. It is worth mentioning that pristine BC usually exhibits a characteristic peak at ~14° [30,45], which is missed in the composite blend. This can be the result of the transformation of cellulose type I to cellulose type II crystalline structure, though most of the articles classify BC as cellulose type I [15,46]. The characteristic peaks of CS are seen at 11.6° and 18.4° [47]. The first peak is assigned to the hydrated crystalline structure of CS while the second one is associated with the amorphous structure of CS [48]. Thus, it can be concluded that through blending of CS macromolecules with the BC semi-crystalline structure, the motion of the host polymer chains is hindered due to the formation of hydrogen bonds, which ultimately results in the disruption of the well-organized BC crystal structure [36]. Introducing ND particles to the polymer matrix results in the appearance of two additional peaks at 2θ = 44° and 75°, which correspond to the (111) and (220) planes of NDs, respectively [39,49]. Meanwhile, the hydrogen bonds arising from the interaction of the CS/BC blend and ND surface functional groups lead to the decrease in crystallinity of the polymeric matrix.

### 2.4. Mechanical Properties

The materials used as wound dressing should fulfill the following mechanical demands: it has to be durable for handling, resistant to the load applied by cells, and conformable to the shape of the body. Besides, it has to be effective in repairing and therapeutic functions [49]. In Table 3, the results of mechanical tests on the composite films are summarized. The elastic modulus obtained (782 ± 20 MPa) is in good agreement with the already reported value of 690 ± 42 MPa [50].

The addition of NDs improved the elastic modulus while the tensile strength was degraded. The enhanced elastic modulus (as high as 3.5- and 4-fold increase upon 2 and 4 wt % of ND, respectively) can be attributed to the intrinsic stiffness of NDs [51] as well as good interfacial adhesion between the nanoparticles and the matrix [52], while lower tensile strength is a result of lower matrix crystallinity [53]. The enhanced elastic modulus parallels with the previous work on poly(methyl methacrylate) where ~2-fold increase upon 5 wt % ND inclusion was observed [54]. Similarly, 5 wt % addition of ND to the PVA matrix resulted in ~3-fold enhancement in the elastic modulus [39].

**Table 3:** Mechanical properties of polysaccharide-based films.

ND (%)	*E* (MPa)	$E_{\text{com}}^{\text{lower}}$ (MPa)	$E_{\text{com}}^{\text{lower}}$ (MPa)	σ_max_ (MPa)	ε_max_ (%)	*K* (kJ/m^3^) × 10
0	782 ± 20	-	-	60.97 ± 0.01	12.53 ± 0.02	0.674 ± 0.000
2	2825 ± 15	961	792000	46.40 ± 1.59	9.77 ± 1.12	0.399 ± 0.005
4	3053 ± 18	783	870000	38.98 ± 2.92	8.27 ± 1.97	0.348 ± 0.004

One may use the Hashin and Shtrikman model [55] to analyze the effect of ND concentration on the elastic modulus of composite films (see Supplementary Materials). The upper and lower values of the composite elastic modulus are reported in Table 3. As seen, the experimental results fall within the predicated value; the differences are attributed to the distribution of the nanoparticles and their interfacial conditions with the polymer matrix. 

**Figure 5:**
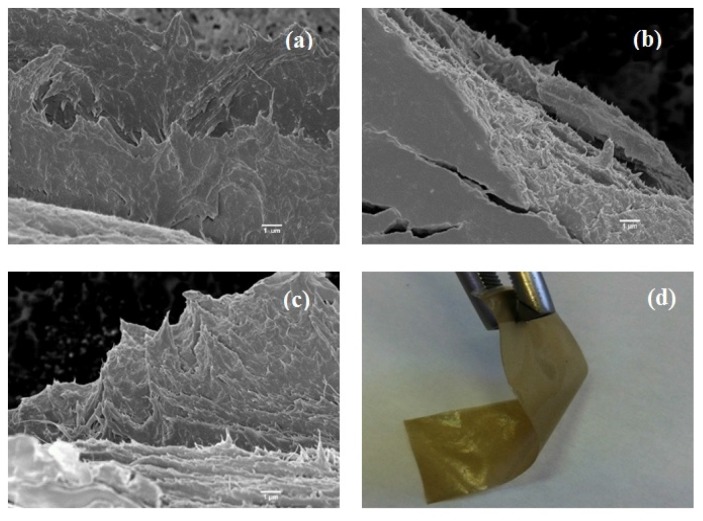
SEM images show the fracture surface of CS/BC films containing (wt %) (**a**) 0, (**b**) 2 and (**c**) 4 NDs. (**d**) Digital image illustrates the flexibility of the nanocomposite films.

The fracture surfaces of tensile tested specimens are shown in Figure 5. Fiber alignment along the applied tensile load and fibers sticking out from the matrix are visible. Sliding of the fibers embedded in matrix could render flexibility (Figure 5d) and an increase in the energy required for the films to fail [56]. 

### 2.5. Cell Viability Assessment

**Figure 6:**
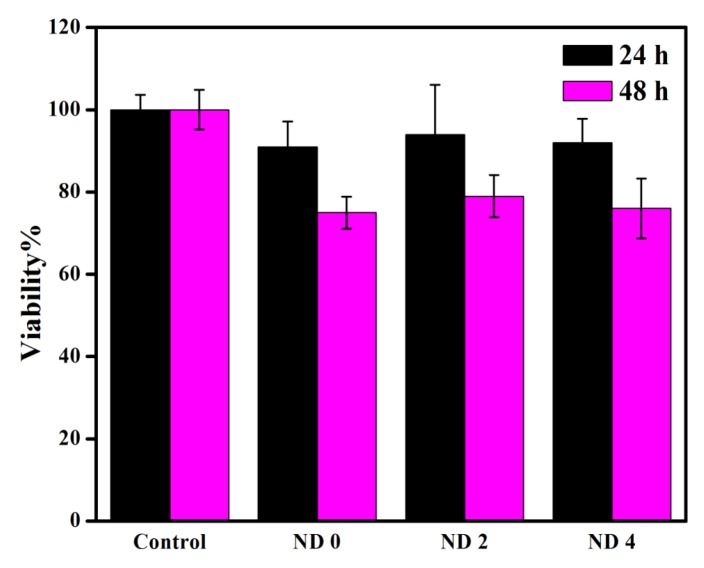
Viability of L929 mouse fibroblast cells incubated on the surface of the composite films.

Although the cytocompatibility of NDs has been established for various cells [57], we employed 3-(4,5-Dimethylthiazol-2-Yl)-2,5-Diphenyltetrazolium Bromide (MTT) assay to evaluate the possible toxicity of the prepared films. The results are summarized in Figure 6. The cell viability of the samples was measured to be more than 90% on the first day and more than 75% on the second day. The results show an improvement over the viability of the L929 cells on the BC/CS films, which has been reported to be approximately 40% after 24 h incubation [31]. This can be attributed to the variability in the preparation step of BC used in this research compared to the previously reported data. Another contributing factor might be the difference in the fabrication step, which potentially could have created more porosities for the support of the cells. Interestingly, the viability is almost maintained for the composite films containing 2 and 4 wt % of ND. The lower viability at high ND concentrations shows a slight cytotoxicity of agglomerated diamond nanoparticles. Although the composites of BC and CS have already been reported in literature [29,31,50], to the best of our knowledge, the incorporation of nanoparticles in this polymeric matrix has not been already tried. This left us with little results to compare our developed system with. 

**Figure 7:**
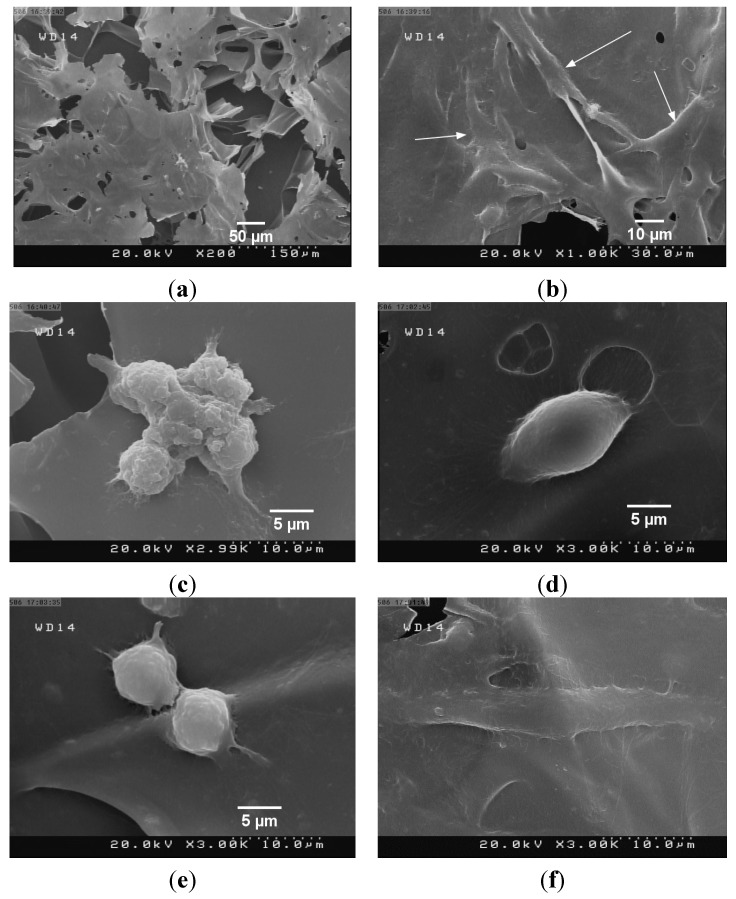
Representative SEM images of composite films. The films were prepared by freeze drying. (**a**) The film surface before cell incubation shows the porous structure of the freeze-dried specimen. Cell morphology on (**b**,**c**) CS/BC and (**d**–**f**) CS/BC/ND (4 wt %) films.

In order to evaluate the attachment of cells on the film surfaces, the freeze-dried specimens were utilized (Figure 7a). It is noteworthy that the micrometric pores in the films may act as templates to guide cell proliferation, differentiation and tissue growth [58]. The images reveal the spindle-like morphology of the cells (Figure 7b,f) as well as the cell expansion (Figure 7d) and cell-to-cell interactions (Figure 7c,e).

It is important to note that cell attachment is a sophisticated process with several stages ranging from the formation of cell binding sites to the activation of the respective signaling pathways, all complicating the analysis. In the case of CS, for example, it has been suggested that the environmental pH, molecular weight, and the degree of deacetylation are amongst the numerous factors that influence the cell attachment behavior [31]. It is also pertinent to point out that for clinical purposes, hemocompatibility, biodistribution, acute toxicity in animal models, and chronic respiration toxicity to major target organs of NDs must be investigated. Previous *in vivo* study [59] has determined that NDs are distributed in the spleen, liver, bones and heart, in addition to the main retention in the lung. Since toxicity of ND is highly dose-dependent, the *in vivo* response and possible safety issues of the films should be evaluated in future.

**Figure 8:**
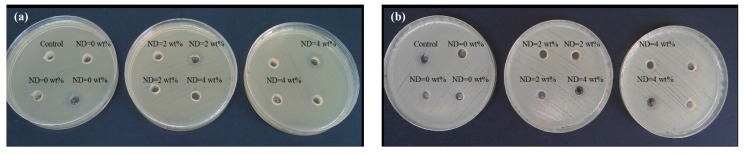
The antibacterial activity of the compounds against (**a**) American Type Culture Collection (ATCC) 25923 *Escherichia coli* and (**b**) American Type Culture Collection ATCC 25922 *Staphylococcus aureus*.

Furthermore, the antibacterial properties of the composite materials were investigated using the Agar well diffusion method in two bacterial strains namely, *Escherichia coli* and *Staphylococcus aureus.* The results are shown in Figure 8. The inhibitory effect of CS against bacteria is well known and is attributed to the interaction of cationic structure of CS with negatively charged moieties on the bacterial cell membrane resulting in the rupture and cell death [60]. Meanwhile, further investigations in terms of antibacterial properties of the compound are required, which would include adopting other routes of antibacterial test such as colony counting method that might be a more suitable option for solid samples such as films with quantitative results [61]. Introduction of ND particles did not exhibit much change in the bactericidal capacity of BC/CS. It has been recently reported that the disappearance of the reactive groups on the surface of ND with bactericidal activity due to the interaction with cellulose membrane can lead to the downregulation of the normal inhibitory effects of as prepared detonation NDs [4].

## 3. Experimental Section 

### 3.1. Materials

Medium molecular weight CS (*M*_w_ = 190–310 kDa, degree of deacetylation: ~85%) was supplied by Sigma-Aldrich Co (St. Louis, MO, USA). Bacterial cellulose nanofibers were purchased from Nano Novin Polymer Co. (Sari, Iran). Nanodiamonds with an average particle size of 5 nm (Grade PL-D-G, purity > 87%) were obtained from PlasmaChem GmbH (Berlin, Germany). Glacial acetic acid was purchased from Merck Co. (Darmstadt, Germany) with analytical grade. 

### 3.2. Sample Preparation

Chitosan/bacterial cellulous/nanodiamond films were prepared by facile solvent casting methods. A chitosan solution was prepared by dissolving 2 g of CS powder in 100 mL acetic acid (1% *v*/*v*). The solution was stirred for 9 h at room temperature and filtered through Wattman No. 41 filter paper (CAT No. 1442-125) to remove the undissolved impurities. Separately, 1 g of BC gel was dispersed in acetic acid (1% *v*/*v*). Aqueous solutions of the individual polymers were mixed at volume ratios of 50/50 and stirred overnight. Nanodiamonds were dispersed in 5 mL deionized (DI) water (Millipore, Billerica, MA, USA, 18 MΩ) through sonication. The suspension was then added to the polymer solution to obtain 1, 2, 3 and 4 wt % of ND suspensions relative to the total dried weight of polymer. The system was stirred for 24 h to obtain a homogenous suspension. After heating and sonication to remove air bubbles, the suspensions (*ca*. 37 mL) were poured into the 10 cm-diameter polystyrene Petri dishes and the solvent was evaporated at room temperature through equally-spaced holes created on the lid.

To improve the binding of the cells to the films, the nanocomposites were also fabricated by freeze-drying method. The samples were cast on the coverslips (18 mm × 18 mm) and were held in a refrigerator at −18 °C for 3 h. The plates were then transferred to a freeze-drying instrument (ALPHA 2–4/LD, Martin-Christ, Osterode am Harz, Germany) where the samples were first dried at −54 °C for 24 h followed by drying at −76 °C for another 6 h under the pressure of approximately 15 Pa. 

### 3.3. Materials Characterization 

#### 3.3.1. Thickness Measurement

The thickness of the films was measured using a digital micrometer (0.001 mm, Absolute Digimatic, Mitutoyo, Tsukuba, Japan). The average of ten points from different regions of the films was determined and reported as the mean film thickness with standard deviation.

#### 3.3.2. Microscopic Studies

The microstructure of the films before and after fracturing was studied by scanning electron microscopy (SEM, Cart Zeiss, Oberkochen, Germany) at an accelerating voltage of 3 kV. The surfaces were carbon sputtered by a metallizer (Quorum Technologies, model Q150ES, Quorum Technologies, East Sussex, UK). To investigate the morphology of the freeze dried and cell-laden samples, field-emission SEM (Hitachi S 4160, Hitachi High Technologies, Tokyo, Japan) was employed. The samples were gold sputtered prior to microscopic observation. Transmission optical microscopy (TOM) examinations were performed using Olympus BX51 optical microscope (Olympus America, Melville, NY, USA) in the transmission mode. 

#### 3.3.3. Optical Properties

The color properties of the films were determined using Color i7 Benchtop Spectrophotometer (XRite, Grand Rapids, MI, USA). Colors were described in the CIELAB space (color space which has been defined by the International Commission on Illumination for describing visible colors as a device independent model) (CIE L*a*b*) by the three typical parameters (L*, a* and b*), where L*, a* and b* indices correspond to lightness, red/green, yellow/blue, respectively. The color coordinates were obtained under illuminate D65 and 2° standard observer. The standard plate was used (L* = 95.38, a* = −0.24 and b* = 2.80) for comparing the parameters of interest. Color difference (∆*E*) was calculated by:

(1) $$∆E=\sqrt{{\left(∆a*\right)}^{2}+{\left(∆b*\right)}^{2}+{\left(∆L*\right)}^{2}}$$

To compare the transparency of the samples, the transmittance profiles were recorded over the range of 360 and 750 nm. 

#### 3.3.4. Attenuated Total Reflectance FTIR

The ATR-FTIR spectra of the composite films were recorded in the transmission mode by utilizing a Bruker Tensor 27 (Bruker Optics Inc., Billerica, MA, USA) with a PIKE ATR Cell accessory (PIKE Technologies, Madison, WI, USA) in the range 550 cm^−1^ to 4000 cm^−1^. 

#### 3.3.5. Thermal Stability and X-Ray Studies

Differential scanning calorimetry (DSC 1/200 System, Mettler Toledo, Greifensee, Switzerland) was undertaken at the heating rate of 10 °C/min under a nitrogen atmosphere using 10 mg of samples over the range of 30–400 °C. The crystallinity of the films was examined by X-ray diffraction (XRD) method. A STOE D-64295 diffractometer (STOE & Cie GmbH, Darmstadt, Germany) using Cu-K_α_ radiation was utilized. The samples were examined over the angular range of 5°−120° with a step size of 0.015°.

#### 3.3.6. Mechanical Measurements

To assess the mechanical properties of the prepared films, the samples were first cut into 10 mm wide and 80 mm long strips. Tensile test was performed using a Universal Testing Machine (Tinius Olsen H10KS, Redhill, UK) equipped with 100 N load cell at a crosshead speed of 1 mm/min. Each test was performed in duplicate. 

#### 3.3.7. *In Vitro* Assessment

Cell viability was evaluated using the standard 3-(4,5-dimethylthiazol-2-yl)-2,5-diphenyl tetrazolium bromide (MTT) assay protocol. The assay is based on the conversion of MTT into formazan crystals by living cells, which determines mitochondrial activity. Briefly, 5 × 10^5^ cells mouse skin fibroblast cells (L929) (National Cell Bank, Iran Pasture Institute) were seeded on the specimens with a 6-well plate and incubated at 37 °C in 5% CO_2_ for 1 and 2 days. After each interval, 200 μL of MTT solution (Sigma, St. Louis, MO, USA, 5 mg/mL) in 1X Dulbecco's Phosphate-Buffered Saline (Sigma, St. Louis, MO, USA) was added to each well and the cells were incubated for another 4 h. Upon removal of the MTT solution, the formed formazan crystals were solubilized with isopropanol for 15 min. Absorbance was read at the wavelength of 570 nm. The data were reported separately for each well by an ELISA reader (BioTek Microplate Reader, BioTek Company, Winooski, VT, USA). An average of triplicate wells were calculated and the standard deviation was calculated for each sample based on Student’s T-test (*p* < 0.05).

To observe the morphology of the adherent cells, the films were washed by DPBS three times and then immersed in 3% glutaraldehyde DPBS solution for 30 minutes for cell fixing. The films were dehydrated in ascending series of ethanol aqueous solutions (50%–100%) at room temperature. The specimens were kept overnight in a desiccator to remove any moisture. The growth of the cell was observed after 24 h of incubation. 

#### 3.3.8. Antibacterial Evaluation

The antibacterial properties of the materials were evaluated against gram-positive and gram-negative bacteria strains, *Staphylococcus aureus* and *Escherichia coli*, respectively. The agar well diffusion assay was adopted where petri dishes (8 mm diameter) were covered with 25 mL of Mueller-Hinton agar with the thickness of 4 mm. The strains were suspended in the sterile saline and diluted at 1 × 10^8^–2 × 10^8^ colony forming unit (CFU)/mL comparable to the turbidity of the 0.5 McFarland standard. The bacteria suspension was inoculated onto the entire surface of the Mueller-Hinton agar plate with a sterile cotton swab to form an even lawn. After agar solidification, wells (6 mm diameter) were punched in the plates using a sterile stainless steel borer. Subsequently, the wells were filled with 75 μL of the samples and were incubated for 24 h at 37 ± 2 °C. The solvent (acetic acid (1% *v*/*v*)) was used as the control. The inhibitory effect of bacteria could be determined by the halo formed around each well. 

## 4. Conclusions 

Flexible and transparent polysaccharide films containing diamond nanoparticles (up to 4 wt %) were fabricated as a potential platform for wound dressing. Effects of NDs on the physiochemical, mechanical and biological properties of the films were studied. The main findings can be summarized as follows:

- A fibrillar-network structure of BC on the surface of the CS films was observed. The distribution of NDs throughout the polymer matrix was uniform at concentrations ≤2%.
- The formation of hydrogen bonds between NDs and the polymer matrix was detected.
- Lower whiteness, higher redness and reduced transparency were obtained when NDs were incorporated into the polymer matrix. Nevertheless, the transparency remained at favorable level due to minimal Rayleigh scattering from the film surface and reasonable ND dispersion.
- A remarkable enhancement in the elastic modulus was obtained by dispersion of NDs in the polymer matrix.
- The addition of NDs reduced the polymer crystallinity, which led to a lower tensile strength.
- Cytotoxic evaluation via culturing of fibroblast L929 cells revealed reasonable cytocompatibility of the composite films containing NDs.
- Examinations of the cell adhesion and interactions revealed the potential of nanocomposite films to support cellular behavior *in vitro*.

## Supplementary Materials

Supplementary materials can be accessed at: http://www.mdpi.com/1996-1944/8/9/6401/s1.
