# Supplementary material for: Development of Chitosan/Bacterial Cellulose Composite Films Containing Nanodiamonds as a Potential Flexible Platform for Wound Dressing
Source: Materials (Basel). 2015 Sep 18;8(9):6401–18. doi: 10.3390/ma8095309 (PMC5512916; doi:10.3390/ma8095309)
Supplement: Supplementary file 1 [file materials-08-05309-s001.pdf]

Supplementary Materials

Estimation of Upper and Lower Bands of Elastic Modulus in Composites

In order to better understand the enhancement effects of nanodiamond articles on the elastic modulus, the model of Hashin and Shtrikman [51] can be used:

$$K_{\mathrm{com}}^{\mathrm{upper}} = K_{\mathrm{f}} + (1 - \varphi_{\mathrm{f}})[\frac{1}{K_{\mathrm{m}} - K_{\mathrm{f}}} + \frac{3\varphi_{\mathrm{f}}}{3K_{\mathrm{f}} + 4G_{\mathrm{f}}}]^{-1} \tag{S1}$$

$$K_{\mathrm{com}}^{\mathrm{lower}} = K_{\mathrm{m}} + \varphi_{\mathrm{f}}[\frac{1}{K_{\mathrm{f}} - K_{\mathrm{m}}} + \frac{3(1 - \varphi_{\mathrm{f}})}{3K_{\mathrm{m}} + 4G_{\mathrm{m}}}]^{-1} \tag{S2}$$

$$G_{\mathrm{com}}^{\mathrm{upper}} = G_{\mathrm{f}} + (1 - \varphi_{\mathrm{f}})[\frac{1}{G_{\mathrm{m}} - G_{\mathrm{f}}} + \frac{6\varphi_{\mathrm{f}}(K_{\mathrm{f}} + 2G_{\mathrm{f}})}{5G_{\mathrm{f}}(3K_{\mathrm{f}} + 4G_{\mathrm{f}})}]^{-1} \tag{S3}$$

$$G_{\mathrm{com}}^{\mathrm{lower}} = G_{\mathrm{m}} + \varphi_{\mathrm{f}}[\frac{1}{G_{\mathrm{f}} - G_{\mathrm{m}}} + \frac{6(1 - \varphi_{\mathrm{f}})(K_{\mathrm{m}} + 2G_{\mathrm{m}})}{5G_{\mathrm{m}}(3K_{\mathrm{m}} + 4G_{\mathrm{m}})}]^{-1} \tag{S4}$$

$$E_{\mathrm{com}}^{\mathrm{upper}} = \frac{9K_{\mathrm{com}}^{\mathrm{upper}}}{1 + 3\,K_{\mathrm{com}}^{\mathrm{upper}}/G_{\mathrm{com}}^{\mathrm{upper}}} \tag{S5}$$

$$E_{com}^{lower} = \frac{9K_{\mathrm{com}}^{\mathrm{lower}}}{1 + 3\,K_{\mathrm{com}}^{\mathrm{lower}}/G_{\mathrm{com}}^{\mathrm{lower}}} \tag{S6}$$

where E_{com} is the elastic modulus of the composite and φ_{f} the volume fraction of the filler. K_{f}, K_{m}, G_{f} and G_{m} are the bulk and shear modulus of the filler and matrix, respectively. Under the assumption that both matrix and filler are isotropic, the following equations are applicable [2]:

$$K_i = \frac{E_i}{3(1 - 2\nu_i)} \tag{S7}$$

$$G_i = \frac{E_i}{2(1 + \nu_i)} \tag{S8}$$

where ν_i is the Poisson's ratio of the filler and/or matrix. The elastic modulus of ND is 967 GPa and its Poisson's ratio is 0.07 [49].
